# Strengthening the enabling environment for women and girls: what is the evidence in social and structural approaches in the HIV response?

**DOI:** 10.7448/IAS.17.1.18619

**Published:** 2014-01-07

**Authors:** Karen Hardee, Jill Gay, Melanie Croce-Galis, Amelia Peltz

**Affiliations:** 1 Formerly Health Policy Project, Futures Group, Washington, DC, USA; 2 J. Gay Consultants LLC, Takoma Park, MD, USA; 3 Artemis Global Consulting, Greater New York Area, NY, USA; 4 United States Agency for International Development, Office of HIV/AIDS, Washington, DC, USA

**Keywords:** women, structural interventions, gender norms, gender-based violence, education, law, livelihoods, stigma and discrimination

## Abstract

There is growing interest in expanding public health approaches that address social and structural drivers that affect the environment in which behaviour occurs. Half of those living with HIV infection are women. The sociocultural and political environment in which women live can enable or inhibit their ability to protect themselves from acquiring HIV. This paper examines the evidence related to six key social and structural drivers of HIV for women: transforming gender norms; addressing violence against women; transforming legal norms to empower women; promoting women’s employment, income and livelihood opportunities; advancing education for girls and reducing stigma and discrimination. The paper reviews the evidence for successful and promising social and structural interventions related to each driver. This analysis contains peer-reviewed published research and study reports with clear and transparent data on the effectiveness of interventions. Structural interventions to address these key social and structural drivers have led to increasing HIV-protective behaviours, creating more gender-equitable relationships and decreasing violence, improving services for women, increasing widows’ ability to cope with HIV and reducing behaviour that increases HIV risk, particularly among young people.

## Introduction

Thirty years into the epidemic, women make up half of those living with HIV. In sub-Saharan Africa young women are as much as eight times more likely than men to be living with HIV [1]. While both men and women are vulnerable to HIV, renewed attention is focussing on ensuring that the factors that drive the unique vulnerabilities of women and girls are addressed in programming. While the AIDS pandemic has shaped a research agenda on gender and the social and structural vulnerabilities of various populations, including women and girls [2], there has been less attention to looking systematically at the interventions to address women’s vulnerabilities, and identifying which interventions have worked is therefore vitally important.

Furthermore, there is growing interest in supplementing public health approaches that focus on individual behaviour to also include approaches that address social and structural drivers, or the environment in which behaviour occurs [3–8]. The Global Prevention Working Group [9] explains that “in addition to individual risk, HIV transmission dynamics are also a function of vulnerability, which stems from social, economic, or legal circumstances that increase susceptibility to infection, deter individuals from seeking essential prevention services, or enhance the likelihood of engaging in unsafe behavior” (p. 9).

The need for interventions that work at the community and societal levels has long been recognized [10]. Structural approaches can be considered to include “social, economic and political interventions that improve public health outcomes by increasing willingness and ability of individuals to practice prevention” (p. 1) [11]. These approaches, undertaken at the distal or upstream level, work through various pathways to affect HIV outcomes [12]. Despite this recognition of the need to address the social and structural drivers of women’s vulnerability to HIV, the Joint United Nations Programme on HIV and AIDS (UNAIDS) highlights “the historic failures of HIV prevention programs to invest in structural interventions” and the need to address legal, economic and social changes “in order to create a more enabling environment for HIV prevention” (p. 25) [13]. Auerbach and colleagues contend that “the arsenal of structural interventions or, more generally, evidence-based and evidence-informed strategies that can be demonstrated to actually achieve social change is quite small” (p. 2) [14]. To address this need and add to the literature, this paper answers the following question: What is the evidence base supporting interventions to address the social and structural drivers and improve the enabling environment for beneficial outcomes for women and girls?

### Defining the enabling environment for women and girls

A number of strategies have been identified over the years to empower women and reduce their vulnerability to HIV. The Global HIV Prevention Working Group lists a number of such interventions, including

legal reform to recognize and protect inheritance and other property rights, microfinance programs and other initiatives to enhance women’s economic independence, universal education for girls, enactment and enforcement of laws against sexual violence, international efforts to eradicate human trafficking, and research initiatives to develop new HIV prevention methods that women can control. Programs to influence the gender norms of men and boys are also essential. (p. 9) [9]

**Figure 1:**
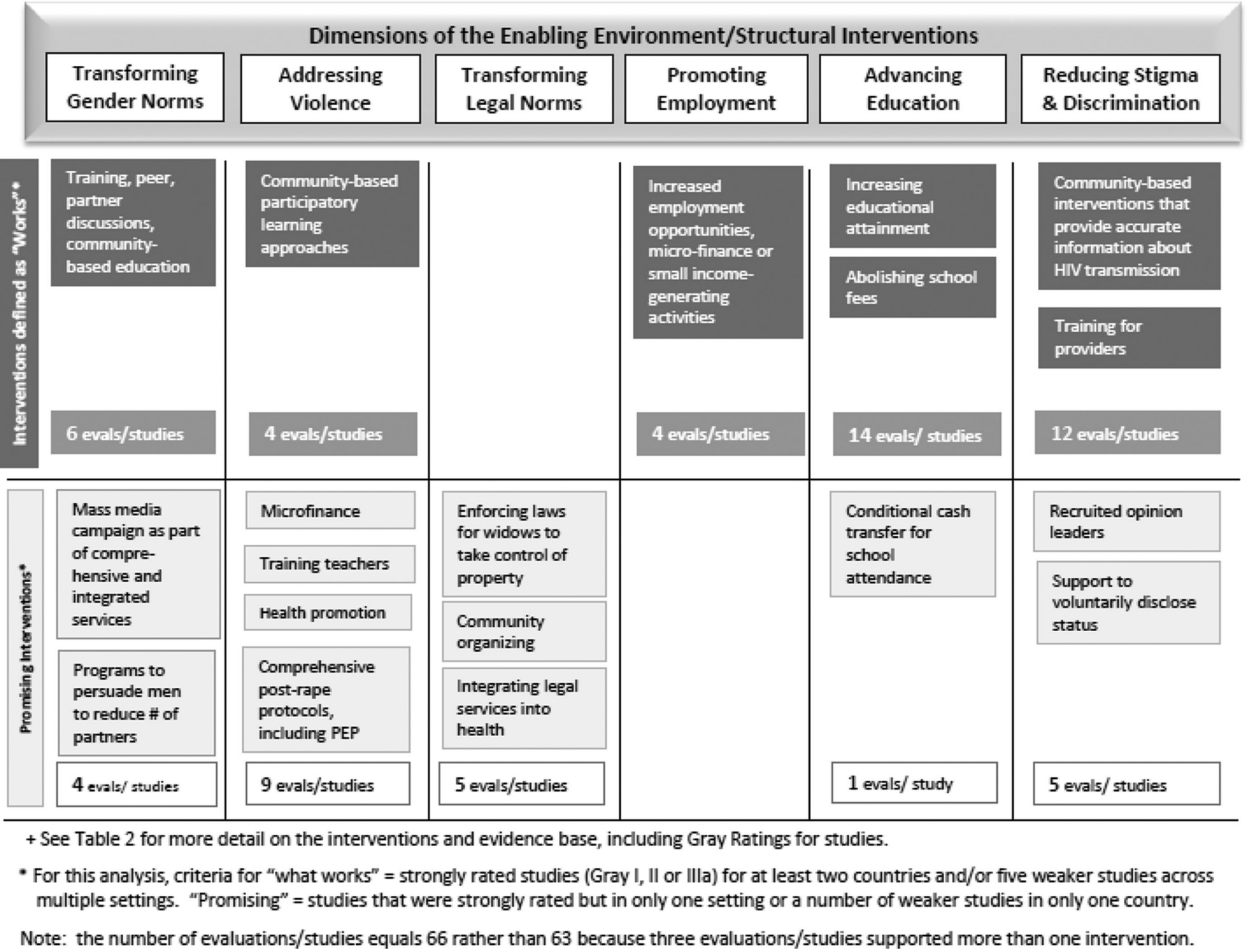
Interventions and supporting evidence for six dimensions of the enabling environment.

Our analysis includes interventions to address five of the social and structural factors noted in this article. Given that both international efforts to eradicate human trafficking and research initiatives to develop new HIV prevention methods that women can control require the most attention at the global level, they are outside of the scope of this review of interventions that can be effective within countries. To these five drivers, we added interventions to reduce stigma and discrimination since addressing this important issue at both societal and structural levels is also critical. Thus, this paper focusses on six key social and structural drivers of HIV vulnerability among women and girls – transforming gender norms; addressing violence against women; transforming legal norms to empower women; promoting women’s employment, income and livelihood opportunities; advancing education for girls and reducing stigma and discrimination (Figure 1) – and reviews the evidence for successful and promising social and structural interventions related to each of these drivers. The analysis in this paper draws from a larger body of work to compile the evidence to inform country-level HIV programming for women and girls regarding which programmes have positive outcomes for women in HIV/AIDS prevention, treatment and care [15].

The paper contends that there are a number of interventions with strong supporting evidence to address these six social and structural drivers to enable women and girls to better reduce their vulnerability to HIV or meet their needs as women living with HIV. Identifying the six social and structural factors does not imply that addressing any one of them alone is sufficient to address the enabling environment; ideally all six will be included in comprehensive programming at the country level.

## Methodology

### Measuring what works: pathways from interventions to outcomes

Measuring the effect on HIV-related outcomes of structural interventions to strengthen the enabling environment is a challenge because “there is no direct, one-to-one relationship between structural interventions and HIV incidence; structural interventions are not generally amenable to randomization; and causal pathways from intervention to AIDS outcome are usually indirect and complex” (p. 1) [11]. Operating in specific socioeconomic, cultural (including gender) and demographic settings, these “distal” or upstream interventions must affect “proximate determinants” such as number of concurrent partners, condom use, blood safety practices and so on, which must act through biological determinants (exposure, efficiency of transmission per contact and duration of infectivity) to affect HIV transmission [16–19]. UNAIDS explains, “Especially for social or structural interventions, where the causal mechanisms may not be widely understood and quantified, it is essential to identify the cascade of results – including the outcomes that each programme component is expected to produce – that links all the elements of the programme in a causal path toward reducing new HIV infections” (p. 14) [13]. In this paper, when interventions are determined to have “worked,” they have been shown to work through a pathway of affecting HIV – or at least a proximate determinant, such as partner reduction or condom use. For each social and structural driver in this paper, the potential causal pathway is described.

### Definition of an intervention

Interventions are defined as programme or policy initiatives that are designed to have a positive effect on improving HIV outcomes for women and girls. Social and structural interventions are designed to address the contextual factors that influence risk behaviour rather than individual risk behaviour. Interventions were not pre-defined; instead, social and structural interventions listed here that aim to strengthen the enabling environment emerged as evidence emerged from the literature to support them. Rather than including entire programmes as successful or not, we determined that understanding the successful component interventions of programmes would be useful for transferring and replicating the interventions and tailoring them to each country’s context. The interventions described here are examples of the types of social and structural interventions for both stand-alone and combination prevention programmes that address biological, behavioural and structural factors [20]. In addition to their own programmatic outcomes, these interventions, taken together, can help to foster an environment that enables HIV programmes and services to achieve greater impact for women and girls.

### Search methodology

This analysis, based on a wider systematic review [15], contains research published in peer-reviewed publications and study reports with clear and transparent data on the effectiveness of interventions related to the six social and structural drivers that can inform programme and policy initiatives to reduce the prevalence and incidence of HIV and AIDS as well as address structural barriers (e.g., legal norms and access to employment) that affect women’s access to prevention, treatment and care services in low- and middle-income countries. Our search strategy focused on the years from 2005 to 2011, using the search words HIV or AIDS and wom*n and other specific terms related to each of the six social and structural drivers. Searches were conducted using SCOPUS, Medline and Popline. This methodology moves beyond the standard of systematic reviews used, for example, by the Cochrane Collaboration [21] because that methodology, while appropriate for clinical practices, is less useful for reviewing evidence on policy and programme interventions. Much of this information comes from unpublished programme reports. Thus, in addition to published literature, the grey literature was captured through review of key websites, including those of UN agencies, the World Health Organization, the Cochrane Collaboration, the Open Society Foundation, Population Services International, the International Center for Research on Women, the Population Council, the International Community of Women, the World Bank, FHI360, AIDStar-One and Two, the Guttmacher Institute, aids2031, and the Global HIV Prevention Working Group. Experts were consulted on each topic to ensure completeness of capturing the evidence.

When an intervention had both positive outcomes and negative outcomes, this was noted. Likewise, we have noted where findings conflict or some studies have shown positive outcomes and other studies have shown negative outcomes. When possible, measures such as a decrease in HIV incidence rates or a decrease in rates of other STIs are used as evidence. If these measures are not available, evidence is drawn from studies measuring externally verifiable measures such as service utilization or, least reliably, self-reported behaviour changes such as condom use, monogamy, sexual abstinence and a decrease in number of sex partners. For many of these, external verification is not feasible and self-reported behaviour is the best available evidence. Where available, we have included sex-disaggregated data. Where an intervention is relevant for both men and women, but does not have sex-disaggregated data, it is identified if included.

One limitation of the methodology used is that the search methodology focussed on health literature; therefore, it may have missed other relevant literature, such as from the disciplines of education and law. For example, increased education for girls is associated with reduced risks of HIV acquisition, yet the search did not include programme interventions to keep girls in school, which would have shown up in the education literature rather than the public health literature. Finally, there are likely many valuable interventions that have not been evaluated and/or published in the public peer-reviewed literature, and important websites may have been unintentionally missed.

### Rating the strength of evidence

Given the breadth of interventions related to HIV programming, which range from clinical treatment to social and structural interventions, a number of systems to rate evidence exist, such as GRADE (Grading of Recommendations Assessment, Development and Evaluation) [22, 23], SORT (Strength of Recommendation Taxonomy) [24], the Center for Evidence-based Medicine’s (CEBM) categorization (www.cebm.net) and HASTE (Highest Available Standard of Evidence); HASTE is a newer system which developed to evaluate programmes for men who have sex with men, and it evaluates interventions based on efficacy data, implementation science data and plausibility [25]. Each system has pros and cons, including its relevance for programmatic versus clinical interventions. For this analysis, the strength of the evidence was rated using a modified version of a classification of strength of evidence termed the Gray scale [26, 27], which was developed for systematic reviews under the Cochrane Collaboration of systematic reviews (http://www.cochrane.org) and lent itself to the variety of interventions in this review. Shown in Table 1, the Gray classification lists five levels of evidence, with the strongest evidence (level I) being systematic reviews of randomized control trials (RCTs) and level V being opinions of respected authorities, descriptive studies or reports of expert committees.

**Table 1:** Gray scale of the strength of evidence, modified

Type	Strength of evidence
I	Systematic review
II	Randomized control trial
IIIa	Study that includes a comparison, or non-equivalent control, group (e.g. quasi-experimental, matched case-control studies or pre-post with control group)
IIIb	Study without a comparison group (e.g. single-group pre-post or cohort)
IV	Qualitative study
V	Opinion of respected authorities, based on clinical evidence, descriptive studies or reports of expert committees.

Note: Gray [26] includes five types of evidence. For this analysis, level III has been subdivided to differentiate between studies and evaluations whose design includes control groups (IIIb) and those that do not (IIIb). Qualitative studies comprise level IV.

Given that RCTs are not always the most appropriate method for studying non-clinical interventions [3, 28], they should not be the only factor used when judging the relative weight of any particular evaluation or study, as Gray [27] notes. Therefore, our methodology incorporates three dimensions: (1) the depth of the evidence (how many evaluations and studies support the intervention), (2) the breadth of the evidence (how many countries contribute evidence to support the intervention), and (3) the strength of the evidence (the Gray scale). Criteria set by expert review committees convened in 2010 and 2011, after much discussion on what level of evidence should constitute “works” versus “promising” given the three criteria established here, were:

- *Works*: Gray I, II or IIIa studies for at least two countries and/or five Gray IIIb, IV or V studies across more than one country.
- *Promising*: Gray I, II or IIIa studies but in only one setting or at least two studies rated Gray IIIb, IV or V in only one country or region.

It is important to note that when an intervention has been shown to work in one setting (internal validity), that does not imply that it will simply also “work” in another setting (external validity). The methodology to determine what works partially addresses internal and external validity by including an element of geographic spread to show the variety of settings in which interventions have been shown to work or to be promising. Still, it is vital that successful interventions be incorporated and adapted for each unique country and programme setting. Knowing that an intervention has worked elsewhere or has shown promising results, and knowing the context in which the intervention was deemed to work or be promising, will help programmes not have to start from scratch in developing interventions.

While gaps in evidence emerged in the course of this research, it was not possible to include examples of interventions that did not have the intended outcomes, largely because research of negative results tends not to be published.

## Results

### Scope of the evidence

The evidence of what works and what is promising among structural interventions to strengthen the six social and structural factors that comprise the enabling environment includes 64 studies and evaluations grouped under 19 interventions (Figure 1). Of those 64 studies and evaluations, 40 support seven interventions that fall under the category of what works, while 24 support 12 promising interventions (Figure 1). The evidence comes from 23 countries, mostly in Africa. Table 2 summarizes the 19 interventions defined by the selection criteria as “works” or “promising,” and the outcomes related to each. The Supplementary file summarizes the interventions and outcomes for the studies and evaluations related to the 19 interventions.

**Table 2:** HIV interventions to strengthen the enabling environment for women and girls by addressing key social and structural drivers, and outcomes, by what works and what is promising

	Intervention	Outcome
**Transforming gender norms**		
W	Training, peer and partner discussions and community-based education	Improve HIV prevention, testing, treatment and care.
P	Mass media campaigns as part of comprehensive and integrated services	Increase HIV protective behaviours.
	Programs to persuade mean to reduce their number of partners	Reduce the risk of HIV acquisition for their female partners.
**Addressing violence against women**		
W	Community-based participatory-learning approaches involving women and men	Create more gender-equitable relationships and decrease violence.
P	Microfinance programs, integrated with participatory training on HIV, gender and violence	Reduction in gender-based violence
	Training teachers about gender-based violence	Change norms about acceptance of gender-based violence.
	Public health promotion	Increase awareness of violence against women.
	Establishing comprehensive post-rape care protocols, which include post-exposure prophylaxis	Improve services for women.
**Transforming legal norms**		
P	Enforcing laws that allow widows to take control of remaining property	Increase widows’ ability to cope with HIV.
	Community organizing to protect rights	Help women pursue their legal rights.
	Integrating legal services into healthcare	Help ensure that women retain their property.
**Promoting women’s employment, income and livelihood opportunities**		
W	Increased employment opportunities, microfinance or small-scale income-generating activities	Reduce behaviour that increases HIV risk, particularly among young people.
**Advancing education**		
W	Increasing educational attainment	Help reduce HIV risk amongst girls.
	Abolishing school fees	Enables girls to attend (or stay in) school,
P	Conditional cash transfers for school attendance	May result in reduced incidence of HIV
**Reducing stigma and discrimination**		
W	Community-based interventions that provide accurate information about HIV transmission	Reduce HIV stigma and discrimination.
	Training for providers along with access to the means of universal precautions	Reduce discrimination against people with HIV.
P	Recruiting and training opinion leaders	Reduce stigmatizing behaviours in the community.
	Support to voluntarily disclose positive serostatus	Increases the ability of people living with HIV to cope and access treatment, and reduces perceived stigma in the community

W=works; P=promising.

### Evidence supporting interventions under the six social and structural factors of the enabling environment

In this section, we describe interventions that were defined in the “works” category and the supporting evidence. More detailed information on these and other promising interventions can be found at http://www.whatworksforwomen.org.

#### Transforming gender norms

The evidence is mounting that gender norms harm both women’s and men’s health [29], but the social issues that women face that make them particularly vulnerable to HIV are related to gender norms that privilege men over women in most societies. Persistent gender-based discrimination and harmful norms that dictate sexual ignorance and submissiveness for females put them at significant risk and create structural barriers to HIV prevention [29–31]. Traditional gender norms lead to behaviours that put everyone at risk for acquiring HIV. Women are less likely to have access to resources and are more likely to depend on men for financial survival for themselves and their children. For many women, having more than one partner is a central survival strategy for themselves and their children. Such dependence makes it difficult to negotiate safer sex with partners.

Interventions to change gender norms are developed on the premise that gender norms, which are passed on by families, peers and institutions, among others, and are interpreted and internalized by individuals, can be changed. Gender relational programming that works with both women and men – in the same or different ways – may be most successful in shifting gender norms in a more equitable direction, with positive impacts on health [32].

Ten evaluations and studies from three continents have demonstrated results for three types of interventions to change gender norms: gender-awareness training, peer and partner discussions and community-based education about changing gender norms (in the “works” category) [33–39]; and mass media campaigns [36, 40] and programmes to change gender norms around multiple partnerships [41, 42] (both “promising”) (see Table 2). An RCT of Stepping Stones for young people in South Africa [33] found that the programme was effective in reducing sexual risk taking and violence perpetuation among young, rural men [34]. Stepping Stones is designed to improve sexual health through building stronger and more gender-equitable relationships among partners, including better communication. Men reported fewer partners, higher condom use and less transactional sex, perpetration of intimate partner violence and substance use [34]. It should be noted that women in the intervention arm had 15% fewer new HIV infections than those in the control arm and 31% fewer herpes simplex virus 2 infections, although neither was significant at the 5% level. Among the women who participated in Stepping Stones, there was an increase in transactional sex.

The One Man Can campaign in South Africa implemented a range of communication strategies, provided training, engaged in advocacy and worked with local government, resulting in men’s increased utilization of voluntary counselling and testing (VCT) and increased use of condoms in addition to more equitable attitudes about gender [35]. Program H in Brazil pioneered programming to change gender norms among young men. A quasi-experimental study, following three groups of young men, tested the hypothesis that young men can change their behaviour and attitudes through participation in group education activities that encourage reflection on what it means to be a man. The intervention also included a community-wide social marketing campaign to promote condom use that used gender-equitable messages. The programme resulted in significantly smaller percentages of young men supporting inequitable gender norms over time [36]. A similar programme in India found that the young men significantly shifted to more gender-equitable attitudes and reported using condoms at last sex [37].

In Tanzania, a community-based HIV and violence prevention programme for young men that combined community-based drama and peer education resulted in significant changes in attitudes and norms related to gender roles and partner violence, as well as increased condom use [38]. A workplace peer group HIV prevention intervention for women in Botswana that addressed issues of gender inequality found that the intervention group significantly increased their HIV prevention behaviours, including personal safer sex practices, positive attitudes toward condoms and confidence in condom use [39].

#### Addressing violence against women

Violence, in addition to being a human rights violation, has been clearly demonstrated as a risk factor for HIV [43–45]. More than one-third of women worldwide experience physical or sexual violence, with regional variations [46]. Violence makes it difficult to negotiate safer sex with partners. Abusive men are more likely to have other sexual partners unknown to their wives [44]. Furthermore, “violence or fear of violence from an intimate partner is an impediment (to) or a consequence of HIV testing” (p. 2) [47]. Children who are sexually abused are more at risk as adults of acquiring HIV [48]. Strikingly, “exposure to violence is already high among young women 15–19, suggesting that violence commonly starts early in women’s relationships” (p. 16) [46].

Thirteen evaluations and studies have shown results for five types of interventions to address violence against women, including community-based participatory learning approaches involving men and women [33 35, 49, 50] (works), and microfinance [51–54], training teachers [55, 56], public health promotion [57–59], and comprehensive post-rape protocols, including post-exposure prophylaxis [60–62] (all promising). Half of the evaluations and studies were conducted in South Africa. Evaluation of the Stepping Stones programme in South Africa found that the programme was effective in reducing sexual risk taking and violence perpetuation among young men. As mentioned, men reported fewer partners, higher condom use and less transactional sex, perpetration of intimate partner violence and substance use [33, 34]. The One Man Can campaign in South Africa resulted in men’s positive attitude shifts regarding gender-based violence [35].

#### Transforming legal norms to empower women, including marriage, inheritance and property rights

Legal norms can enhance or hinder HIV programming and the delivery of effective and equitable services. For both women and men, gender norms are codified through public policy in a range of issues [29, 63, 64]. For example, laws can and often do reinforce the subordinate status of women by denying women the right to divorce, the right to own property and the ability to enter into contracts****, to sue and testify in court****, to consent to medical treatment and to open a bank account, all of which affect the legal rights of women [65, 66] These restrictions can make a woman less likely to leave an abusive situation that may place her at risk of HIV acquisition. In many of the countries where women are most at risk for acquiring HIV, laws to protect women are weak [65–67]. In some countries, people living with HIV have little access to the formal legal system [68], even though women living with HIV particularly need knowledge of their rights [69] to counter discrimination.

Five evaluations and studies, all from Africa, have shown results for three types of interventions to transform legal norms, all of which fall in the promising category. These interventions include enforcing laws that allow widows to take control of remaining property [70, 71], community organizing to protect rights [72, 73] and integrating legal services into health services [74].

#### Promoting women’s employment, income and livelihood opportunities

Economic independence may not always have a clear-cut role in HIV acquisition, with both wealth and poverty being associated with risks and protective effects for HIV acquisition, depending on the different contexts [75]. Yet women’s economic dependence on men and unequal access to resources, including land and income-generating opportunities, have been shown to increase the likelihood of women and girls engaging in a variety of unsafe sexual behaviours, including transactional sex, coerced sex, earlier sexual debut and multiple sexual partners, and thus increase their risk of becoming infected with HIV [76, 77]. Economic dependence also drives women to accept men’s multiple partnerships [78]. Independent sources of income and employment for women may allow women to insist on safe sex [79] and to refuse sex to men who refuse to wear condoms [80].

Four evaluations and studies have shown results for one type of intervention that works: increased employment opportunities such as microfinance and small-scale income-generating activities [Any discussion or consideration of microfinance programmes should note that these programmes could also increase violence against women if the intervention is not carefully designed and appropriate to the local context [4, 85, 86].] can reduce behaviour that increases HIV risk, particularly among young people, in certain circumstances [81–84]. The evidence comes from Africa and the Caribbean. Analysis from the Intervention with Microfinance for AIDS and Gender Equity (IMAGE) study in South Africa found that after two years of follow-up, young women who had received microfinance loans to establish small businesses, along with training on gender and HIV, were more likely to have accessed VCT and less likely to have had unprotected sex at last intercourse, and they were more likely to have had communication concerning HIV with sexual partners and others [81]. A time-usage study that analysed data on education, work and organized activities among young people in South Africa found that employment opportunities decreased the odds of sexual activity among girls and that higher wages for both boys and girls were associated with increased condom use [82].

In Haiti, a microfinance project that provided loans to women living with HIV as well as HIV-negative women found that the loans had improved their life conditions and that loan repayment was high [83]. Four years after an income generation and HIV prevention project for youth was initiated in the Republic of Congo, a follow-up inquiry found that 24% of the youth were still involved in income-generating activities which, for the girls especially, reduced their dependency on others, although mobility and exposure to non-familiar adults in insecure forms of activity may counter some of these beneficial effects [84].

#### Advancing education

UNICEF contends that “ensuring quality education for all children is one of the best ways to protect both the rights and the lives of young people threatened by HIV/AIDS” (p. 2) [87]. Analysis by the Global Campaign for Education estimates that seven million HIV infections in young people could be averted in a decade if all children completed primary school [88]. Analysis of data across countries and educational levels shows that “young women and men with higher levels of education are more likely to have increased knowledge about HIV/AIDS, a better understanding of ways to avoid infection, and an increased likelihood of changing behaviour that puts them at risk of contracting the disease” (p. 2) [87]. The effectiveness of education as an HIV prevention strategy, which the World Bank calls the “window of hope,” rests on greater access to schooling and on using schools to reach young people with HIV education and life skills training [89]. Yet girls face barriers to staying in school, including lack of money needed for school funds, uniforms, textbooks and supplies, as well as pressure from their parents to marry [90].

Furthermore, lack of sanitary facilities has been hypothesized to influence girls’ and teachers’ school attendance during menstruation [91].

Fifteen studies, including multicountry analyses, demonstrate the strong link between increasing educational attainment and reduction in risk of HIV [88, 92–102] (works), along with abolishing school fees [103, 104] (works) and conditional cash transfers [105] (promising). For example, multivariate survival analysis in South Africa showed that one additional year of education reduced the hazard of acquiring HIV by 7% net of sex, age, wealth, household expenditure, residence, migration status and partnership status [94]. A 2009 study in Ethiopia, Ghana, Kenya, Malawi and Mozambique showed that fee abolition resulted in an average 23% increase in total enrolment across the five countries [103].

Conditional cash transfers represent a promising approach to keeping girls in school and having positive outcomes on HIV prevention [105]. This approach is considered “promising” in this analysis, even though an RCT in Malawi found positive effects of school attendance, because that is the only study found to support the intervention and thus it does not yet rise to the “works” category. More evidence on conditional cash transfers will certainly emerge in the near future.

#### Reducing stigma and discrimination

Stigma and discrimination have been identified as tremendous barriers to addressing HIV/AIDS [106–110]. Stigma affects prevention behaviours, test seeking, care seeking, the quality of care provided to clients living with HIV, and perceptions and treatment of people living with HIV and AIDS by communities and families [108]. Women are often considered to face the double stigma and discrimination associated with HIV and their inferior status to men in society [111–114]; women living with HIV often must “balance the stigma of being HIV-positive with the reality that childbearing is often their only route to social status and economic support” (p. 51) [111]. Yet, many studies of stigma and discrimination do not collect sex-disaggregated data, making it difficult to determine the differential experiences that men and women face [108].

Seventeen evaluations and studies, most from Asia, have shown results for four types of interventions to reduce stigma and discrimination: community-based interventions that provide accurate information about HIV transmission [115–121] (works); training for providers, along with access to universal precautions [122–126] (works); recruiting and training opinion leaders [127, 128] (promising); and providing support to voluntarily disclose positive serostatus [109, 129, 130] (promising). An intervention in two communities in Vietnam, where stigma was so strong that no one was open about their HIV status, led to a significant increase in awareness of stigma and reduction in fear of becoming infected with HIV through casual contact with HIV-positive people [115].

In Malawi, a national mass media campaign – including radio diaries featuring the lives of a man and a woman living with HIV, radio programming educating youth about HIV and community mobilization using trained community facilitators – resulted in a significant positive association between programme exposure and being less likely to show stigmatizing attitudes to people living with HIV. This, in turn, was associated with an increased uptake of HIV testing [121].

Training for providers, including emphasis on safe healthcare practices, can reduce discrimination against people with HIV in healthcare settings, as shown in Vietnam [122], India [123] and China [124]. For example, training for service providers in county hospitals in Yunnan, China, resulted in a stronger belief in patient confidentiality, reduced fear of people living with HIV and better knowledge and practice of universal precautions [124]. Interventions with dental students in Turkey [125] and nurses in five African countries [126] also showed reductions in stigmatizing and discriminatory attitudes and behaviour.

## Discussion and recommendations

There are increasing calls to address the structural drivers of HIV as part of comprehensive responses to the epidemic. Seeley et al. [7], in a special issue of this journal, note that addressing structural drivers of HIV should not be considered a “luxury” in programmes facing declining resources, saying that “initiatives to achieve HIV elimination will only come about through a comprehensive HIV response, that includes meaningful responses to the social, political, economic and environmental factors that affect HIV risk and vulnerability.” Yet there has been insufficient attention to programming to address structural factors, including the structural factors that women face. Rao Gupta and colleagues [131] contend that “no country has yet achieved impact on gender-related HIV vulnerability at sufficient scale to change the course of the AIDS epidemics they face” (p. S370).

This paper reviewed the evidence generated from structural interventions to address the six social and structural factors that constitute part of the enabling environment. The evidence shows that educating girls, and providing them with employment or income-generating opportunities, in an environment with equitable gender norms, respect for legal rights, non-acceptance of violence, and freedom from stigma and discrimination, will have beneficial HIV-related outcomes for women and girls. Successful interventions use a number of approaches, including training for individuals, communities and providers; community-based participatory learning and education for males and females; peer support; mass media campaigns; financial services and social protection programmes linked with training on gender, HIV and violence reduction; law enforcement and greater legal literacy; and promoting school attendance by girls, including by abolishing school fees. These approaches have led to increasing HIV-protective behaviours; creating more gender-equitable relationships and decreasing violence; improving services for women; increasing widows’ ability to cope with HIV; reducing behaviour that increases HIV risk, particularly among young people; enabling girls to attend (or stay in) school and reducing HIV stigma and discrimination. In addition, a number of promising approaches exist that have shown positive results in one setting or through less rigorous studies in a range of settings. While no intervention included in this paper has addressed all six social and structural drivers together, nevertheless, knowing the evidence base for each of the six drivers is important to strengthen the enabling environment for strong, comprehensive HIV programmes. Programmes using this evidence to enhance their own structural interventions to address these social and structural drivers of HIV among women will clearly need to adapt the programming to their own contexts. Scaling up these structural interventions will require an understanding of the sociocultural and programmatic contexts in each country, [9, 132–134], particularly for the reason that “because the humanistic, participatory and gender-sensitive components of an innovation are the most difficult to replicate on a larger scale, they are often the first to be sacrificed” (p. 42) [135].

For each of the six social and structural factors included in this paper, many gaps in programming and research remain. While strong evidence exists for some components, such as girls’ education, there is still a dearth of evidence related to some components of the enabling environment, including legal reform and enhancing employment and economic opportunities. Given that the AIDS epidemic is in its third decade, it is unfortunate that addressing the social and structural factors has been given relatively little attention in the AIDS response and that the evidence base is not deeper. For example, the importance of transforming gender norms in increasing women’s ability to negotiate safer sex practices is clear, although there is insufficient evidence on how to change norms on a national scale.

Further research to expand the evidence base of the range of social and structural factors considered part of the enabling environment is warranted. In order to continue building the evidence base of successful interventions that work to strengthen the enabling environment, greater efforts are needed to research, translate and bring to scale promising social and structural interventions. Recognizing that RCTs are not always possible or appropriate for these types of interventions, there is a need for greater investment in implementation science [136] to develop robust and interdisciplinary methods and tools to evaluate social and structural interventions that strengthen the enabling environment. The application of rigorous social science methods to the development and testing of tools to identify and measure outcomes that serve as good HIV proxies or measures of social change is essential, particularly when evaluating social and structural interventions.

Furthermore, this paper shows that while there is geographic spread of the evidence on interventions to address social and structural drivers of HIV for women, there is a need to test and evaluate social and structural interventions to strengthen the enabling environment in a range of countries and epidemiological contexts. For example, South Africa predominates in studies on violence against women, while most studies on stigma and discrimination have taken place in Asia.

Ultimately, halting and reversing the HIV epidemic, particularly among women and girls, will not be possible without addressing – on a large scale – these underlying factors that put women and girls at greater risk. Restrictive gender norms, violence, legal inequalities, stigma and discrimination, and unequal access to economic opportunities and education lie at the heart of women’s greater HIV risk. All of these structural factors can be addressed through proven and promising interventions, scaled up within each country’s social and programmatic context.
